# Fatherhood group sessions: A descriptive and summative qualitative study

**DOI:** 10.1111/nhs.12776

**Published:** 2020-10-04

**Authors:** Anita Berlin, Michael Rosander, Karin F. Frykedal, Lena Törnkvist, Mia Barimani

**Affiliations:** ^1^ The Division of Nursing, Department of Neurobiology, Care Sciences and Society Karolinska Institutet Stockholm Sweden; ^2^ Psychology, Department of Behavioral Sciences and Learning Linköping University Linköping Sweden; ^3^ Department of Social and Behavioural Studies University West Trollhättan Sweden; ^4^ Education, Department of Behavioral Sciences and Learning Linköping University Linköping Sweden; ^5^ Department of Neurobiology, Care Sciences and Society Karolinska Institutet Stockholm Sweden; ^6^ Academic Primary Care Centre Stockholm Sweden; ^7^ Department of Learning, Informatics, Management and Ethics (LIME) Karolinska Institutet Stockholm Sweden

**Keywords:** child health, infants, leader roles, new fathers, parental groups, Sweden

## Abstract

The aim of this qualitative study of fatherhood group sessions offered as part of child health care services for new parents was to examine the activities, roles, and topics initiated by the leader and describe fathers' participation. Eight new fathers took part in three audio‐ and video‐recorded sessions led by a male leader. Three qualitative content analysis approaches were used to analyze the data. The analysis showed that the group leader took on four leadership roles, mainly that of discussion leader, but also expert, friend, and organizer. When the group leader acted as discussion leader, fathers participated by discussing challenges and changes in their new situation. Challenges were related to raising the child, partner relationships, everyday life, and gender equality. Fathers also discussed changes in their partner relationships and an increased focus on practicalities in daily life. Fatherhood groups can help new fathers form social networks and can create space for fathers to work through challenging topics, such as gender equality in parenting. The discussion leader's choice of role is crucial to creating the space for such discussions.

## 1 INTRODUCTION

Becoming a father is a life‐changing event that causes a mixture of positive and challenging emotions (Kowlessar, Fox, & Wittkowski, 2015; Ngai & Lam, 2020; Sansiriphun, Kantaruksa, Klunklin, Baosuang, & Liamtrirat, 2015). Qualitative studies that give new fathers a voice have revealed that many go through emotional challenges throughout the transition to parenthood, including feeling detached from their partner's pregnancy experience and questioning their role (Åsenhed, Kilstam, Alehagen, & Baggens, 2014; Kowlessar et al., 2015). In the early days after the birth, men can experience fatherhood as a period of trial and error and helplessness (Kowlessar et al., 2015). Additionally, Swedish fathers have reported difficulty managing the demands of fatherhood, poor emotional health, and a lack of time for their partner, child, friends, and personal interests (Johansson, Thomas, Hildingsson, & Haines, 2016). In fact, many Swedish fathers feel that having a baby was in some ways less than a positive experience (Johansson et al., 2016). Additionally, more than a third of 827 fathers surveyed reported financial and health worries, concerns about achieving balance in their new situation, and apprehension about raising a child (Johansson et al., 2016). Swedish fathers described the postnatal period as a “roller coaster” of emotion that includes both stress and feelings of great joy (Åsenhed et al., 2014, p. 1309).

## 2 BACKGROUND

Support can help reduce worries and anxiety during the transition to parenthood (Barimani, Vikström, Rosander, Forslund Frykedal, & Berlin, 2017; Bryanton, Beck, & Montelpare, 2013). Group‐based parental training or parental education groups (hereafter PE groups) are one way to provide support and prepare expectant and new parents for childbirth and parenting (Barimani et al., 2017; Gagnon & Sandall, 2007). Parents who attend such groups often find it reassuring to meet others in similar circumstances and satisfying to have the opportunity to establish social networks (Berlin, Törnkvist, & Barimani, 2016). However, they can feel dissatisfied with PE groups' primary focus on childbirth and lack of discussion about parenting and changes in partner relationships (Berlin et al., 2016). Moreover, a lack of group activities means that parents' wishes to interact with other parents are not always fulfilled (Berlin et al., 2016; Forslund Frykedal & Rosander, 2015). When group leaders take on the role of experts and give lectures, it also leaves little time for parents to actively participate and interact (Berlin, Rosander, Forslund Frykedal, & Barimani, 2018).

PE groups in Sweden typically include both mothers and fathers (National Handbook of Child Health Services, 2020a) and are led by midwives or nurses in child health care services (hereafter CHC services), most of whom are women (Barimani et al., 2017). A higher percentage of women than men participate in PE groups (Hallberg, Beckman, & Håkansson, 2010; Petersson, Petersson, & Håkansson, 2003), and men often perceive that support is primarily focused on women (Petersson et al., 2003). Fathers attending mixed‐gender PE groups may feel “invisible, disregarded and insulted” (Salzmann‐Erikson & Eriksson, 2013, p. 381) and that the content preserves traditional gender roles (Berlin et al., 2016). Fathers can also find it difficult to know what questions to ask during PE groups (Erlandsson & Häggström‐Nordin, 2010).

As part of health care services for new parents, CHC services have started to offer fatherhood group sessions at some centers. However, these remain less frequent than mixed‐gender sessions. There is thus a shortage of studies examining what fatherhood group sessions can offer fathers. This is an important question, as traditional PE groups seem to fail to provide support for new fathers (Kowlessar et al., 2015) and it is children's right to have equally prepared and supported parents (Eklund & Lundqvist, 2018).

We had the unusual opportunity to observe and record several fatherhood group sessions that were offered through CHC services and held by a male leader. In this qualitative study, we aimed to observe and describe (i) the activities, roles, and topics initiated by the leader; and (ii) new fathers' participation in the sessions.

The analysis focused on information that could answer the following questions:

1. What leader activities and roles can we identify?
2. How much time did the leader devote to different roles during the sessions?
3. What topics did the leader bring up in his different roles and how did the fathers respond?
4. How much time did the leader and participants spend on different activities during the sessions?

## 3 METHOD

### 3.1 Design

The study had a qualitative, descriptive, and summative design as outlined by Hsieh and Shannon (2005) and used audio and video recordings and observations. We chose this design to obtain access to the phenomenon under study (fatherhood groups) in a naturalistic setting (CHC services). Such access is difficult for approaches that rely on qualitative interviews and/or quantitative methods (Salmon, 2015).

### 3.2 Study setting

Swedish family policy has a critical influence on parenthood and on the topics discussed in PE groups; it is therefore an important aspect of the study setting. In Sweden, gender equality goals are emphasized in national, government‐led, public health awareness programs (Government Office of Sweden, 2008a; Swedish Social Insurance Agency, 2018a, 2018b). Over the years, political discussions, as well as policy discussions in public institutions such as the CHC services, have focused on how to support and enable parents to take equal responsibility for their children (Government Office of Sweden, 1997, 2008a, 2008b). Fathers' involvement in their children's lives promotes children's mental health and social adaptation, and taking equal responsibility for children seems to benefit parent–child attachment (Sarkadi, Kristiansson, Oberklaid, & Bremberg, 2008; World Health Organization [WHO], 2007).

Early parenting support in PE groups is offered to all new parents in Sweden and their children (National Handbook of Child health Services, 2020a). These groups are free of charge and mostly led by nurses from CHC services (Government Office of Sweden, 2008b). PE groups normally consist of 5–15 new parents. A group starts when the child is 6–8 weeks old and meets regularly during the child's first year (National Handbook of Child Health Services, 2020a). Whereas approximately 93% of new mothers participate in PE groups, only 6% of fathers take part (Child Health Care Unit, Stockholm County, 2019). Reasons for fathers' low participation may include the tendency to hold PE groups during the workday and/or paternal experience of the CHC services environment as non‐inclusive and unwelcoming (Wells, Engman, & Sarkadi, 2015).

If fathers express an interest in fatherhood group sessions, centers sometimes offer them though contracts with private companies. However, group sessions targeting new fathers are not a routine part of CHC services. Discussion in the CHC services has focused on the possibility that unisex group sessions could enhance rather than lessen gender role differences in parenting (National Handbook of Child Health Services, 2020a). No support for this hypothesis has been found in the scientific literature.

### 3.3 Participants

To recruit participants, we provided the administrative office of a county council in an urban area with verbal and written information about the study. We arranged meetings with health care developers at the Department of CHC services to find a fatherhood group leader and obtain his or her contact information. The health care developers provided information on a leader, and one of the authors contacted him by email. He was a father who had previously led fatherhood group sessions in urban areas in Sweden.

After receiving verbal and written information about the study, the group leader and eight fathers agreed to participate. Some fathers brought their infants (age range 7 weeks to 6 months) to the sessions. The participating fathers (*N* = 8, age range 28–44 years) were all biological parents of the children. One father had an upper secondary school education, two were university students, and five had completed university. Two were born in the Middle East and the rest in Sweden. For six fathers, this was their first child, and for the rest, it was their second. Seven cohabitated with their partners, and one lived alone. The participants met for three sessions that adhered in content to the national goals for PE groups: helping parents prepare for the transition to parenthood, increasing knowledge about child development, facilitating co‐parenting relationships, and developing social networks (National Handbook of Child health Services, 2020b). At the first session, the fathers and the leader agreed to discuss expectations, relationships, and family life at the sessions.

### 3.4 Data collection

Four observational approaches to data collection were used: (i) *audio recordings* (all three sessions), (ii) *video recording* (one session), (iii) direct *observation* with *field notes* (all three sessions), and (iv) verbatim *transcripts* of the three audio recordings. In qualitative research, observational methods provide a thorough description of participants and their activities. Furthermore, they are suitable for capturing the natural setting of clinical practice (Patton, 2002; Salmon, 2015).

Audio‐ or video‐recorded material, observations, and field notes were collected by two of the authors for 5 weeks in April and May 2015, and the audio and video recordings were transcribed by one of the authors. The recordings were made with a single camera and an audio recorder positioned prior to the commencement of the sessions. Recorded material varied in length (90–105 min/session) and comprised a total of 4 h and 45 min (1 h and 30 min of video and 3 h 15 min of audio recording; Table 1). The video recording helped capture how the leader behaved when performing different activities but could only be used at one session for practical and ethical reasons. Two researchers attended each group session and sat at the back of the room with the intention of causing as little disturbance as possible. One researcher was responsible for the audio and video recorders and the other took field notes throughout the sessions to complement the recorded material. The notes focused on the number of participants, activities in the room, topics presented, and questions asked.

**TABLE 1:** Participants, data collection, and duration of three group sessions for new fathers

Fatherhood group session	Session 1	Session 2	Session 3
		After 3 weeks	After 2 weeks
Number of fathers	8	6	6
Number of infants	1	4	3
Session duration	2 h	2 h	2 h
Type of recording	Audio^a^	Video/audio	Audio^b^
Video or tape duration	105 min	90 min	90 min

^a^ One of the participants declined to be video recorded.

^b^ Video camera not available.

### 3.5 Data analysis

We used three approaches to qualitative content analysis outlined in Hsieh and Shannon, (2005): conventional (inductive category development), directed (further analysis using the inductively developed categories), and summative (counting/calculating events and activities in audio and video recordings). The analysis was performed in four steps. In the first step, we used a conventional approach to categorize leader activities and roles in the audio and video material (research question 1). To analyze these data, the recordings were uploaded to the software program MAXQDA11 (MAXQDA, 2019), designed for computer‐assisted qualitative and quantitative data analysis. The first author (A.B.) marked segments in which the leader took an active role (i.e. initiated or performed certain activities). Using MAXQDA11 and the transcripts, she wrote descriptions of activities and employed an inductive approach to create codes and categories for different activities and roles. In addition, she consistently noted quotes that related to these different activities and roles. A.B. initially analyzed two audio recordings. This initial analysis was sent to M.R. and K.F.F. for checking. After they confirmed the analysis, A.B. analyzed the remaining data but did not find any new leader activities or roles. During the conventional analysis, A.B. continuously compared the content of observations and field notes with the audio‐ and videotapes of the group sessions to check for consistency. In the second step, A.B. employed the directed approach, using the inductively developed categories of leader behavior, activities, and roles as a point of departure and focusing on identifying the topics that the leader initiated when he took on each role (research question 3). In this step, she again viewed the video and listened to the audio recordings. In a third step, A.B. used a conventional approach to categorize the content relevant to fathers' verbal responses in the transcripts (research question 3). She identified two categories: *change* and *challenges*. In a fourth step, she used the summative approach to count the duration of different activities and roles (research question 2) and determine the proportion of leader activity and time allowed for fathers' responses (research question 4). The duration of different leader activities was calculated with MAXQDA11. After each step, M.R. and K.F.F. checked and confirmed the analyses.

### 3.6 Ethical considerations

Throughout the study, we adhered to the ethical principles of the British Psychological Society (2014): respect, competence, responsibility, and integrity. We obtained written informed consent from the group leader and the fathers. All participants could decline to be video and/or audio recorded. One father declined to be video recorded but accepted audio recording, so when he was present, the cameras were shut off (see Table 1). The research project was approved by the regional Research and Ethics Committee at Linköping University, Sweden (reference # 2013/401–31).

## 4 RESULTS

### 4.1 Leader activities and roles

In the analysis of audio and video recordings, we identified leader behaviors and activities that characterized four roles: *discussion leader*, *expert*, *friend*, and *organizer* (Table 2). The leader engaged in each role to a different degree.

**TABLE 2:** Leader activities and leader roles based on actions taken by the leader in fatherhood group sessions

Codes	Categories
Leader activities	Leader roles
Asks questions	Discussion leader
Allocates different tasks	
Creates commitment and involvement	
Confirms	
Demonstrates or guides during different tasks	
Gives space to father responses	
Initiates activities	
Examines expectations	
Gives information, mediates certain kinds of knowledge	Expert
Gives practical advice/suggestions	
Shares personal experience and ideas	Friend
Makes jokes	
Shows concern, understanding, and empathy	
Creates lists of names and addresses of participants	Organizer
Is welcoming	
Opens and closes the session	
Presents the aim of the group session	
Presents the theme of the day	
Sets timeframes: Total number of sessions, times to meet	
Provides coffee breaks	
Initiates evaluation	
Initiates planning for future independent meetings of the group	

#### 4.1.1 Discussion leader

The leader assessed expectations, initiated discussions on agreed‐upon topics and homework assignments, evaluated fathers' opinions, and strove to create intragroup trust by stressing the importance of confidentiality: “What's said here stays in the group!”

In addition, the leader asked reflective questions and encouraged the fathers to listen to each other and share their experiences. For instance, he said, “It's good that we listen to each other and share our experiences. It's a give and take!”

#### 4.1.2 Expert

The leader referred to science when talking about worries and difficulties that women and men experience during parenthood. He contributed expert knowledge related to scientific studies, such as national and international statistics about the length and gender distribution of parental leave: “The social insurance system gives Swedish parents the right to 480 days of paid parental leave. Despite the goal of equality, estimates show that 75% of parental leave is used by mothers, while fathers/partners take 25%.”

#### 4.1.3 Friend

The leader made statements and sometimes joked about shared personal experiences, ideas, and perceptions. One example of this kind of statement was “This is what my wife and I did in similar situations.”

#### 4.1.4 Organizer

In this role, the leader dealt with practical matters such as scheduling (e.g. opening and closing each session, planning for coffee breaks). He offered a list of contact information to participants, gave homework assignments, and initiated planning for independent group meetings: “Does the group intended to continue meeting? Is one of you willing to be responsible for that?”

### 4.2 Distribution of leader activity and participant time

In the first and the last group sessions, the leader spent most of his time – 35% to 50% – in his role as discussion leader. The fathers had the opportunity to speak between half and two‐thirds of the total time at these sessions (see Table 3).

**TABLE 3:** Percentages of leader role/activity and participant time in the sessions

Leader role/activity	Session 1	Session 2	Session 3	Total
Discussion leader	65%	30%	43%	45%
Expert	14%	36%	29%	27%
Friend	13%	16%	14%	14%
Organizer	8%	18%	14%	14%
Total leader activity	34%	48%	39%	40%
Total participant activity	66%	52%	61%	60%
Total time	*105 min*	*92 min*	*90 min*	*287 min*

#### 4.2.1 Leader role, topics initiated, and fathers' response

The agreed‐upon topics that the leader initiated at each session were: Session 1 – expectations, the child's personality, relationship to the child, and caring for a child; Session 2 – partner relationships, parental roles, common conflicts, and parental leave; and Session 3 – achieving balance in life (including work‐life balance), family, partner time, individual free time, and sleep. Lectures and conversations were the main teaching techniques. The role that the leader adopted and the topic he presented led to differing responses from the fathers. Responses ranged from quiet listening to active participation via discussing, reflecting, making statements, and asking questions (Table 4).

**TABLE 4:** Descriptions of leader roles, topics initiated, and fathers' responses

Leader roles	Topics initiated	Fathers' responses
Discussion leader	Caring for and raising a child	Discussing Raising questions
	Relationship to partner	Discussing
	Gender equality in parenthood	Discussing Making statements
	Relations with the extended family	Discussing Reflecting
Expert	UN Children's convention, the child's human rights, parent and adult responsibility for a child	Quiet listening: no verbal response, no questions raised
	Paid parental leave Gender distribution of parental leave (percent per partner) Question from leader: Is the use of parental leave decided by chance or by pre‐existing circumstances?	Discussing Making statements Raising questions
Friend	Everyday matters	Laughing, making brief comments
Organizer	Traditional PE groups (PE groups provided for both men and women at CHC services) Future independent group meetings of the group	Discussing Making statements

Abbreviations: CHC, child health care; PE, parental education.

### 4.3 The content of fathers' verbal responses

In their verbal responses to topics initiated by the leader, fathers took up perceived challenges and changes (Table 5).

**TABLE 5:** Categories, subcategories, and codes drawn from data gathered during three fatherhood group sessions

Category	Subcategory	Codes
Challenges	Managing and raising a child	Interpreting the child's personality Sleeping and feeding problems Anxiety for health and well‐being
	Handling the couple relationship	Troublesome communication Conflicts Reduced time for their partner Shortage of sleep Unspoken expectations
	Managing everyday life	Managing planning Establishing routines Achieving a balance Feelings of stress and a bad conscience
	Managing aspects of gender equality	Differences between mothers and fathers Use of parental leave Attending traditional PE groups on equal terms
Changes	Becoming more connected	A “project” in common Closer to relatives
	A different focus	Practicalities dominate life

Abbreviation: PE, parental education.

### 4.4 Challenges

Fathers discussed several challenges, such as how to manage and raise their child, how to handle their relationship with their partner, everyday life, and aspects of gender equality.

#### 4.4.1 Managing and raising a child

Challenges that fathers brought up included how to provide the best care for their child, how to raise their child, and how to interpret and understand their child's personality. The fathers discussed the anxiety they felt about their child's overall health and well‐being when the child was not eating or sleeping properly. They asked questions such as the following during the discussion: “How is it possible to manage everything when you have a second child?” (Father 1), and “How is it possible to raise a baby girl to become a strong and independent individual?” (Father 2).

#### 4.4.2 Handling the couple relationship

Fathers discussed how challenging it was to get communication with their partners to work and described how this difficulty led to conflicts almost every day: “We have disputes about minor things. We never had that many conflicts before!” (Father 3). Fathers could also perceive that their partners had unspoken expectations. It was especially hard to handle these expectations when they and their partners were short on sleep and did not have enough time with each other: “Your partner expects you to be able to interpret thoughts, needs, and feelings!” (Father 3).

One participant said that his partner had gone through a lot of physical challenges during pregnancy and childbirth. He therefore stressed the importance of showing understanding, paying attention, and being gentle.

#### 4.4.3 Managing everyday life

Fathers brought up a number of everyday challenges, such as achieving balance in life. Balancing family life, working life, and personal time caused constant feelings of stress and a bad conscience at home and at work. One father explained, “I never know if I'm doing the right thing. I have bad conscience since I'm not at home together with them [the mother and child]. At work, I feel stressed by the need to get home as soon as possible” (Father 1). Other challenges included structuring life, managing planning, solving everyday problems, and getting routines to work: “Before we were structured, we had a plan and could get things done. But all that collapsed when the baby came” (Father 5).

#### 4.4.4 Managing aspects of gender equality

Some fathers brought up their belief that there is a difference between mothers and fathers. They described mothers as comforters, the ones who know the routines and what the child needs (clothing and other material goods) and fathers as entertainers, the ones who make funny faces to get the child to laugh.

It was challenging to take equal responsibility for the child and household work. One father stressed the importance of avoiding the traditional man's trap of working longer than normal. The men then discussed the dangers of the traditional woman's trap, taking full responsibility for the child and housework. “I have female friends who are active feminists fighting for equality between women and men,” said one participant. “After they had their children, they stayed at home, took most of the responsibility, and used all of the paid parental leave [did not divide the paid parental leave equally with their partners]” (Father 2).

The fathers accepted that given the importance of breastfeeding, it would be hard for them to take the first part of parental leave. However, they stressed the need for parents to make equal use of parental leave in order to spend equal time with their child. They brought up a number of factors outside their immediate control that made it hard to achieve this goal, including social structure, family budget, and salaries. One said, “The way things turn out depends on the social structure in society. It's common for women to take the majority [of parental leave], since in general their salary is lower” (Father 6).

All the participating fathers had attended traditional PE groups with their partners as part of antenatal care. They discussed the advantage of continuing to attend such groups, which might promote the exchange of information, experiences, and opinions with other mothers and fathers. Moreover, it would also provide an opportunity to ask a health care professional questions about a variety of topics, which could reduce parental anxieties and worries. One father joked that if you attend a PE group, “You don't need to tire out your friends with questions and worries regarding your child” (Father 4). On the other hand, one father problematized attendance: “Once my partner and I attended a PE group at the CHC services. I was the only man there. I felt different, uncomfortable, and unequal” (Father 6).

The fathers expressed a willingness to continue holding independent fatherhood group meetings since they felt it was good to meet and discuss aspects of fatherhood.

### 4.5 Changes

Fathers discussed changes that had come with parenthood, such as changes to the couple relationship. The most significant change in the fathers' relationship with their partners was becoming closer and more connected because of their joint parenthood “project.” They also explained that their relationship had shifted to focus on everyday practicalities, which they perceived as dominating life. One father stated that his partner had changed. “She's always here and now. When [the baby] has just fallen asleep, I want to take advantage of it, relax and slow down…. Then she says: lay him down and start making dinner. She's become so practical” (Father 3).

The fathers also described a stronger connection to their immediate and extended families. For example, after becoming parents they regarded meeting and celebrating holidays together with the extended family as more fun. One father said, “With a child, the Christmas celebration will change – there will be more joy and fun. Santa Claus can visit again” (Father 4).

## 5 DISCUSSION

Audio and video recordings revealed that the leader took on four different roles. When he adopted the role of discussion leader, he created time and space for discussions. In this role, he also strove to create intragroup trust and the prerequisites for active and open response by stressing the importance of confidentiality and the value of sharing experiences. Fathers responded by discussing challenges and changes in their new situation. The results showed that the topics and activities in the fatherhood group sessions followed the national guidelines for PE groups (National Handbook of Child health Services, 2020b). Topics included the child's overall development, the couple relationship, everyday life, co‐parenting relationships, and aspects of gender equality in parenthood. Fathers also made plans for future independent meetings, which indicates that they had succeeded in developing a social network, an important goal of PE groups (National Handbook of Child health Services, 2020b).

The findings of this study suggest that the discussion leader role may be crucial in promoting the goals of PE groups. This finding is consistent with those of a previous study of PE groups, which found that when group leaders take on the role of experts and give lectures, parents have little room to actively participate and interact (Berlin et al., 2018).

A growing body of evidence supports the hypothesis that fatherhood group sessions are helpful to fathers. A recent review of parental education concludes that men‐only classes can be useful for supporting new fathers (Lau & Hutchinson, 2020, p. 4). Discussing fatherhood from a male perspective may reduce concerns and struggles earlier reported by fathers, such as how to best support their families emotionally, physically, and financially (Johansson et al., 2016). Fathers in a former study reported that discussing emotions from a male perspective gave them insight and inspired them to further prepare for fatherhood (Pålsson, Persson, Ekelin, Kristensson Hallström, & Kvist, 2017). Such group sessions may also be positive for participants' children. When fathers are involved in parenting, it improves their attachment to their child, which in turn has positive effects on the child's overall health and well‐being (WHO, 2007). A 2020 integrated review found that group sessions for fathers, offered in the community, were the most effective intervention for improving fathers' involvement with their children and thereby promoting child development (Henry, Julion, Bounds, & Sumo, 2020). Thus, our findings and those of other previous studies contrast with recommendations from CHC services that problematize single‐gender group sessions (National Handbook of Child Health Services, 2020b). This component of the guidelines should therefore be reconsidered.

Fathers in this study discussed the challenge of gender equality and differences in men's and women's parenting roles. It was clear that they were aware that such inequalities persist despite national and public health programs promoting gender equality. The understanding of gender equality and gender differences in parenting is still in its infancy (Doucet, 2009, p. 117), and this topic deserves further attention from researchers. Another topic for further investigation is how much the gender of the group leader impacts fathers' willingness to discuss their concerns. Previous studies are suggestive but not conclusive on this point. A study of fatherhood‐only discussions, led by a man (Pålsson et al., 2017, p. 90), found that these sessions provided fathers with an opportunity to discuss fatherhood. In another study, men leading fatherhood group sessions explained that it was important to them to get fathers to reflect on parenthood and to empower their role in the family (Kerstis, Wells, & Andersson, 2018). On the other hand, the role that the group leader chooses to assume – for example, discussion leader or expert – may be of equal or greater importance (Berlin et al., 2018). Studies comparing fatherhood groups led by men and women could help resolve this question.

### 5.1 Methodological considerations

To the best of our knowledge, the real‐life setting of fatherhood group sessions has not previously been studied using video and/or audio material. The main strength of this method of data collection is direct access to the phenomenon under study, which might be difficult to obtain with qualitative interviews and/or quantitative methods (Salmon, 2015). Research guidelines (Swedish Research Council, 2017) stress that video recordings can negatively impact participants' integrity. In response, we informed participants about the recording ahead of time and gave them the opportunity to decline recording without any impact on the group session or their participation. When carried out in this manner, recording is an open approach in which participants are clearly aware that they are being observed and is ethically sound (van Deventer, 2009). It remains possible that recording or the presence of observers influenced the group leader's performance and/or the fathers' participation.

The concepts of credibility, confirmability, and transferability describe different aspects of trustworthiness (Graneheim & Lundman, 2004). To achieve credibility, the research team discussed the video and audio data, as well as the analysis, until we reached consensus about codes and categories. To attain confirmability, we added quotes to illustrate the descriptions of the group leader's roles and the fathers' responses. Fewer quotes were used to illustrate the group leader's role as friend, as many relevant quotes would have revealed personal information.

We attempted to facilitate transferability by providing a clear description of the study context, data collection, and data analyses. The structure and goals of fatherhood groups differ between countries, so findings from the Swedish context might not be transferable elsewhere. However, the results about the leader's role seem less context‐bound and may therefore be worth further research in other contexts.

## 6 CONCLUSIONS AND IMPLICATIONS

This was a descriptive and summative qualitative study of eight fathers in one urban area in Sweden, which limits our ability to draw conclusions. Nevertheless, the findings show that the role of discussion leader is crucial to achieving open discussions in a fatherhood group setting. The study also adds to our understanding of fathers' thoughts and concerns about topics covered in national PE group guidelines, such as co‐parenting relationships. Finally, it adds to the growing body of international literature in support of fatherhood groups. Such groups can create time and space for discussions on aspects of fatherhood that new fathers find important. They can help fathers work through parenting topics, including but not limited to gender equality in parenting. Moreover, these groups can achieve other policy goals, such as helping new fathers establish supportive social networks. It may be time to reconsider policies that advise against such groups.

## CONFLICT OF INTEREST

No conflict of interest has been declared by the authors.

## AUTHOR CONTRIBUTIONS

All authors acquired funding and collected audio and video data. A.B., with help from M.R. and K.F.F., designed, analyzed, and categorized data. A.B. drafted the manuscript and M.R., K.F.F., M.B., and L.T. critically revised the manuscript and approved the final version.

## ETHICS STATEMENT

The research project was approved by the Regional Research and Ethics Committee at Linköping University, Sweden (reference # 2013/401–31).

## AUTHOR STATEMENT

All authors meet the criteria for authorship, have contributed significantly, and have approved the final article.
